# Dichlorido(3,5,5′-trimethyl-1,3′-bi-1*H*-pyrazole-κ^2^ *N* ^2^,*N* ^2′^)copper(II)

**DOI:** 10.1107/S1600536811004375

**Published:** 2011-02-12

**Authors:** Lhoussaine El Ghayati, Lahcen El Ammari, Mohamed Labd Taha, El Mostafa Tjiou

**Affiliations:** a Laboratoire de Chimie Organique Hétérocyclique, Pôle de Compétences, Pharmacochimie, Av Ibn Battouta, BP 1014, Faculté des Sciences, Université Mohammed V-Agdal, Rabat, Morocco; b Laboratoire de Chimie du Solide Appliquée, Faculté des Sciences, Université Mohammed V-Agdal, Avenue Ibn Battouta, BP 1014, Rabat, Morocco; c Laboratoire de Chimie Bio Organique Appliquée, Faculté des Sciences, Université Ibn Zohr, Agadir, Morocco

## Abstract

In the title complex, [CuCl_2_(C_9_H_12_N_4_)], the Cu^II^ atom exhibits a distorted square-planar coordination geometry involving two chloride ions and two N-atom donors from the bipyrazole ligand. The chelate ring including the Cu^II^ atom is essentially planar, with a maximum deviation of 0.0181 (17) Å for one of the coordinated N atoms. This plane forms a dihedral angle of 30.75 (6)° with the CuCl_2_ plane. In the crystal, each pair of adjacent mol­ecules is linked into a centrosymmetric dimer by N—H⋯Cl hydrogen bonds. The crystal structure is stabilized by inter­molecular C—H⋯N and C—H⋯Cl hydrogen bonds and weak slipped π–π stacking inter­actions between symmetry-related mol­ecules, with an inter­planar separation of 3.439 (19) Å and a centroid–centroid distance of 3.581 (19) Å.

## Related literature

For the preparation of biheterocyclic complexes, see: Juanes *et al.* (1985 ▶); Arrieta *et al.* (1998 ▶); El Ghayati *et al.* (2010 ▶); Cohen-Fernandez *et al.* (1979 ▶); Tarrago *et al.* (1980 ▶). For applications of transition metal complexes with biheterocyclic ligands, see: Bekhit & Abdel-Aziem (2004 ▶); Benabdallah *et al.* (2007 ▶); Das & Mittra (1978 ▶); Sendai *et al.* (2000 ▶); Attayibat *et al.* (2006 ▶). 
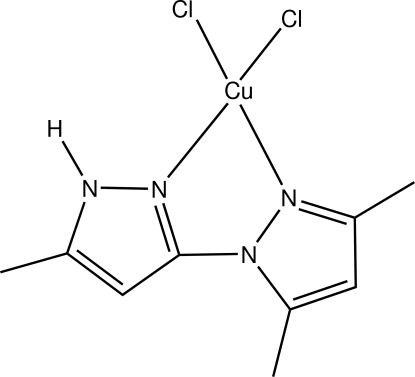


## Experimental

### 

#### Crystal data

[CuCl_2_(C_9_H_12_N_4_)]
*M* *_r_* = 310.67
Triclinic, 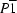

*a* = 8.5475 (2) Å
*b* = 9.3475 (3) Å
*c* = 9.3512 (3) Å
α = 66.379 (2)°
β = 62.876 (1)°
γ = 78.065 (2)°
*V* = 608.99 (3) Å^3^
*Z* = 2
Mo *K*α radiation
μ = 2.21 mm^−1^
*T* = 296 K
0.26 × 0.16 × 0.08 mm

#### Data collection

Bruker X8 APEXII area-detector diffractometer
Absorption correction: multi-scan (*SADABS*; Bruker, 2005 ▶) *T*_min_ = 0.661, *T*_max_ = 0.838
19588 measured reflections
5535 independent reflections
4468 reflections with *I* > 2σ(*I*)
*R*_int_ = 0.020

#### Refinement

*R*[*F* ^2^ > 2σ(*F* ^2^)] = 0.030
*wR*(*F* ^2^) = 0.096
*S* = 1.04
5535 reflections
145 parameters
H-atom parameters constrained
Δρ_max_ = 0.78 e Å^−3^
Δρ_min_ = −0.53 e Å^−3^

### 

Data collection: *APEX2* (Bruker, 2005 ▶); cell refinement: *SAINT* (Bruker, 2005 ▶); data reduction: *SAINT*; program(s) used to solve structure: *SHELXS97* (Sheldrick, 2008 ▶); program(s) used to refine structure: *SHELXL97* (Sheldrick, 2008 ▶); molecular graphics: *ORTEP-3 for Windows* (Farrugia,1997 ▶) and *PLATON* (Spek, 2009 ▶); software used to prepare material for publication: *WinGX* (Farrugia, 1999 ▶).

## Supplementary Material

Crystal structure: contains datablocks I, global. DOI: 10.1107/S1600536811004375/fj2386sup1.cif

Structure factors: contains datablocks I. DOI: 10.1107/S1600536811004375/fj2386Isup2.hkl

Additional supplementary materials:  crystallographic information; 3D view; checkCIF report

## Figures and Tables

**Table 1:** Hydrogen-bond geometry (Å, °)

*D*—H⋯*A*	*D*—H	H⋯*A*	*D*⋯*A*	*D*—H⋯*A*
N4—H4⋯Cl1^i^	0.86	2.38	3.1587 (12)	150
C7—H7*B*⋯N1^ii^	0.96	2.61	3.483 (2)	151
C9—H9*B*⋯Cl1^iii^	0.96	2.79	3.5377 (19)	135

Symmetry codes: (i) 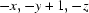; (ii) 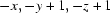; (iii) 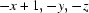.

**Fig. 1.**
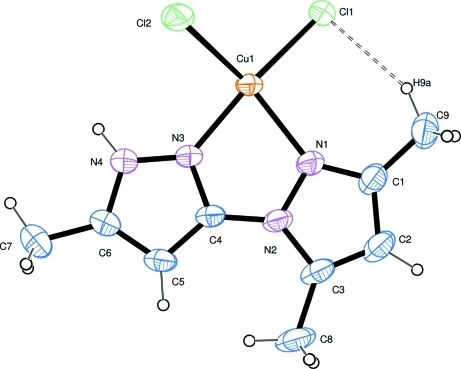
The asymmetric unit of the title compound, with the atom-labeling scheme. Displacement ellipsoids are drawn at the 50% probability level. H atoms are represented as small spheres of arbitrary radii.

**Fig. 2.**
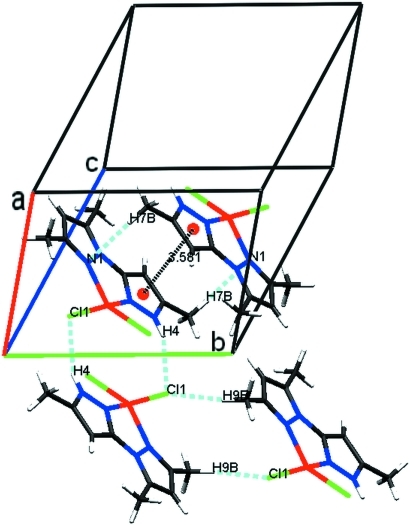
Packing diagram showing hydrogen-bonded (dashed lines) complex molecules and distance between centroids.

## supplementary crystallographic information

### Comment

The 1,1'-bipyrazoles and 3,4'-bipyrazoles have been the subject of several studies (Juanes *et al.* (1985); Arrieta *et al.* (1998); El Ghayati *et al.* (2010). A particular interest has been brought to 1,3'-bipyrazoles which present, contrary to those cited above, a carbon– nitrogen bond between the two pyrazoles (Cohen-Fernandez *et al.* (1979); Tarrago *et al.* 1980).

The ability of biheterocycles to form biochemically interesting complexes, with transition metals has prompted several researchers to test them in some areas: medicine (Bekhit & Abdel-Aziem, (2004); Sendai *et al.* 2000), agriculture (Das & Mittra, 1978) corrosion (Benabdallah *et al.* 2007) and as extractors of metals such as Cu^2+^, Cd^2+^ and Pb^2+^ (Attayibat *et al.* 2006). To better understand the interactions between the bipyrazoles and transition metals we have chosen to study some copper complex of bipyrazole possessing a Carbone-nitrogen bond between the two pyrazolics cycles.

The title molecule is built up from two interconnected five-membered rings as schown in Fig.1. Each of the two heterocyclic rings and the linked carbon are almost planar with a maximum deviations of -0.0101 (15) Å and -0.0107 (15) Å from N1 and N3 respectively. The dihedral angle between them is about 3.80 (9)°. The Cu^II^ ion is surrounded by two nitrogen atoms belonging to the organic molecule and two chlorides which form a very distorted square planar.The values of adjacent angles around the Cu^II^ ions are in the range 78.14 (5)–98.297 (16)° and 151.99 (4)–161.72 (4)° (Table 1), which confirms the distorted square-planar geometry. The chelate ring (N1—N2—C4—N3) and the copper atom are almost planar with a maximum deviations of 0.0181 (17) Å from C4 and build dihedral angle of 30.75 (6)° with the plane through the three ions: Cu^II+^ and two Cl^-^.

In the crystal, each pair of molecules linked by N4—H4···Cl1 hydrogen bonds form a dimer as schown in Fig.2 and table 2. The structure is held together by weak slipped π-π stacking between symmetry related molecules (N3—N4—C4—C5—C6 rings) with interplanar distance of 3.439 (19) Å and centroid to centroid vector of 3.581 (19) Å (Fig. 2). The crystal structure is also stabilized by an intermolecular C7—H7B···N1 and C9—H9B···Cl1 hydrogen bonds as schown in Fig.2 and Table 2.

### Experimental

The title compound was synthesized by mixing a solution of bipyrazole in methanol and an aqueous solution of cupric chloride with ligand/metal ratio of 2. Heating was maintaind for few minutes.Then a pinch of NaCl was added and heating was continued until the solution became clear. After a long time, green crystals were collected and dried over P2O5.

### Refinement

The C-bound H atoms were positioned geometrically [C—H = 0.93–0.96 Å] and refined using a riding model with *U*_iso_(H) = 1.2 and 1.5 for methylene and methyl. Reflections 2–43 110, 250, 3–21, 114 and 1–31 were omitted because of the large difference between their calculated and observed intensities.

### Crystal data

**Table:**

[CuCl_2_(C_9_H_12_N_4_)]	*Z* = 2
*M**_r_* = 310.67	*F*(000) = 314
Triclinic, *P*1	*D*_x_ = 1.694 Mg m^−^^3^
Hall symbol: -P 1	Mo *K*α radiation, λ = 0.71073 Å
*a* = 8.5475 (2) Å	Cell parameters from 5535 reflections
*b* = 9.3475 (3) Å	θ = 2.9–35.5°
*c* = 9.3512 (3) Å	µ = 2.21 mm^−^^1^
α = 66.379 (2)°	*T* = 296 K
β = 62.876 (1)°	Prism, clear green
γ = 78.065 (2)°	0.26 × 0.16 × 0.08 mm
*V* = 608.99 (3) Å^3^	

### Data collection

**Table:**

Bruker X8 APEXII area-detector diffractometer	5535 independent reflections
Radiation source: fine-focus sealed tube	4468 reflections with *I* > 2σ(*I*)
graphite	*R*_int_ = 0.020
φ and ω scans	θ_max_ = 35.5°, θ_min_ = 2.9°
Absorption correction: multi-scan (*SADABS*; Bruker, 2005)	*h* = −13→13
*T*_min_ = 0.661, *T*_max_ = 0.838	*k* = −15→15
19588 measured reflections	*l* = −15→15

### Refinement

**Table:**

Refinement on *F*^2^	Primary atom site location: structure-invariant direct methods
Least-squares matrix: full	Secondary atom site location: difference Fourier map
*R*[*F*^2^ > 2σ(*F*^2^)] = 0.030	Hydrogen site location: inferred from neighbouring sites
*wR*(*F*^2^) = 0.096	H-atom parameters constrained
*S* = 1.04	*w* = 1/[σ^2^(*F*_o_^2^) + (0.053*P*)^2^ + 0.1288*P*] where *P* = (*F*_o_^2^ + 2*F*_c_^2^)/3
5535 reflections	(Δ/σ)_max_ = 0.001
145 parameters	Δρ_max_ = 0.78 e Å^−^^3^
0 restraints	Δρ_min_ = −0.53 e Å^−^^3^

### Special details

**Table:**

Geometry. All s.u.'s (except the s.u. in the dihedral angle between two l.s. planes) are estimated using the full covariance matrix. The cell s.u.'s are taken into account individually in the estimation of s.u.'s in distances, angles and torsion angles; correlations between s.u.'s in cell parameters are only used when they are defined by crystal symmetry. An approximate (isotropic) treatment of cell s.u.'s is used for estimating s.u.'s involving l.s. planes.
Refinement. Refinement of *F*^2^ against ALL reflections. The weighted *R*-factor *wR* and goodness of fit *S* are based on *F*^2^, conventional *R*-factors *R* are based on *F*, with *F* set to zero for negative *F*^2^. The threshold expression of *F*^2^ > 2σ(*F*^2^) is used only for calculating *R*-factors(gt) *etc*. and is not relevant to the choice of reflections for refinement. *R*-factors based on *F*^2^ are statistically about twice as large as those based on *F*, and *R*- factors based on ALL data will be even larger.

### Fractional atomic coordinates and isotropic or equivalent isotropic displacement parameters (Å^2^)

**Table:**

	*x*	*y*	*z*	*U*_iso_*/*U*_eq_	
Cu1	0.15845 (2)	0.378309 (18)	0.126139 (19)	0.03371 (6)	
Cl1	0.32602 (6)	0.27845 (5)	−0.07595 (5)	0.04576 (10)	
Cl2	0.17371 (7)	0.63132 (4)	−0.04198 (5)	0.05123 (11)	
N1	0.19189 (18)	0.19578 (13)	0.32890 (15)	0.0350 (2)	
N2	0.06621 (18)	0.19922 (13)	0.48517 (14)	0.0338 (2)	
N3	−0.03865 (17)	0.41525 (14)	0.32360 (14)	0.0341 (2)	
N4	−0.16369 (17)	0.52865 (15)	0.34407 (15)	0.0357 (2)	
H4	−0.1782	0.6055	0.2609	0.043*	
C1	0.2969 (2)	0.07094 (17)	0.3644 (2)	0.0415 (3)	
C2	0.2341 (3)	−0.00505 (18)	0.5432 (2)	0.0471 (4)	
H2	0.2824	−0.0952	0.6008	0.056*	
C3	0.0881 (3)	0.07866 (16)	0.61715 (19)	0.0406 (3)	
C4	−0.05733 (19)	0.32244 (15)	0.48068 (15)	0.0304 (2)	
C5	−0.1967 (2)	0.37413 (18)	0.60475 (17)	0.0368 (3)	
H5	−0.2360	0.3297	0.7234	0.044*	
C6	−0.26295 (19)	0.50668 (17)	0.51088 (18)	0.0344 (2)	
C7	−0.4133 (2)	0.6144 (2)	0.5670 (2)	0.0456 (3)	
H7A	−0.4235	0.6954	0.4680	0.068*	
H7B	−0.3936	0.6602	0.6326	0.068*	
H7C	−0.5199	0.5571	0.6366	0.068*	
C8	−0.0262 (4)	0.0550 (2)	0.8008 (2)	0.0576 (5)	
H8A	−0.1176	0.1347	0.8090	0.086*	
H8B	0.0429	0.0607	0.8553	0.086*	
H8C	−0.0779	−0.0457	0.8567	0.086*	
C9	0.4575 (3)	0.0306 (3)	0.2294 (3)	0.0605 (6)	
H9A	0.4677	0.1029	0.1184	0.091*	
H9B	0.4495	−0.0735	0.2372	0.091*	
H9C	0.5591	0.0362	0.2453	0.091*	

### Atomic displacement parameters (Å^2^)

**Table:**

	*U*^11^	*U*^22^	*U*^33^	*U*^12^	*U*^13^	*U*^23^
Cu1	0.04382 (11)	0.02877 (8)	0.02047 (8)	0.00351 (6)	−0.00980 (7)	−0.00738 (5)
Cl1	0.0559 (2)	0.04221 (17)	0.02692 (14)	0.01764 (15)	−0.01377 (14)	−0.01382 (13)
Cl2	0.0665 (3)	0.03027 (15)	0.03254 (17)	−0.00083 (15)	−0.00632 (17)	−0.00494 (12)
N1	0.0460 (7)	0.0321 (5)	0.0255 (5)	0.0042 (4)	−0.0167 (5)	−0.0094 (4)
N2	0.0466 (7)	0.0307 (5)	0.0221 (4)	−0.0015 (4)	−0.0159 (4)	−0.0052 (4)
N3	0.0410 (6)	0.0351 (5)	0.0211 (4)	0.0044 (4)	−0.0124 (4)	−0.0083 (4)
N4	0.0386 (6)	0.0383 (5)	0.0261 (5)	0.0048 (4)	−0.0128 (4)	−0.0112 (4)
C1	0.0589 (10)	0.0325 (6)	0.0408 (7)	0.0091 (6)	−0.0303 (7)	−0.0141 (5)
C2	0.0759 (12)	0.0306 (6)	0.0423 (8)	0.0058 (6)	−0.0376 (8)	−0.0085 (5)
C3	0.0649 (10)	0.0296 (5)	0.0295 (6)	−0.0066 (6)	−0.0262 (7)	−0.0023 (5)
C4	0.0375 (6)	0.0313 (5)	0.0207 (5)	−0.0060 (4)	−0.0111 (4)	−0.0062 (4)
C5	0.0420 (7)	0.0409 (6)	0.0217 (5)	−0.0071 (5)	−0.0074 (5)	−0.0096 (5)
C6	0.0332 (6)	0.0398 (6)	0.0288 (6)	−0.0058 (5)	−0.0075 (5)	−0.0147 (5)
C7	0.0378 (8)	0.0488 (8)	0.0470 (9)	−0.0006 (6)	−0.0081 (6)	−0.0253 (7)
C8	0.0937 (16)	0.0430 (8)	0.0262 (6)	−0.0065 (9)	−0.0249 (8)	−0.0007 (6)
C9	0.0735 (14)	0.0597 (11)	0.0552 (11)	0.0337 (10)	−0.0389 (11)	−0.0298 (9)

### Geometric parameters (Å, °)

**Table:**

Cu1—N3	1.9496 (12)	C2—H2	0.9300
Cu1—N1	2.0707 (11)	C3—C8	1.485 (2)
Cu1—Cl1	2.2106 (4)	C4—C5	1.396 (2)
Cu1—Cl2	2.2456 (4)	C5—C6	1.385 (2)
N1—C1	1.3436 (18)	C5—H5	0.9300
N1—N2	1.3720 (17)	C6—C7	1.488 (2)
N2—C3	1.3552 (17)	C7—H7A	0.9600
N2—C4	1.3935 (18)	C7—H7B	0.9600
N3—C4	1.3260 (16)	C7—H7C	0.9600
N3—N4	1.3453 (17)	C8—H8A	0.9600
N4—C6	1.3431 (18)	C8—H8B	0.9600
N4—H4	0.8600	C8—H8C	0.9600
C1—C2	1.406 (2)	C9—H9A	0.9600
C1—C9	1.486 (3)	C9—H9B	0.9600
C2—C3	1.375 (3)	C9—H9C	0.9600
			
N3—Cu1—N1	78.14 (5)	N3—C4—C5	111.28 (12)
N3—Cu1—Cl1	161.72 (4)	N3—C4—N2	114.03 (12)
N1—Cu1—Cl1	97.11 (3)	C5—C4—N2	134.67 (12)
N3—Cu1—Cl2	93.55 (4)	C6—C5—C4	104.26 (12)
N1—Cu1—Cl2	151.99 (4)	C6—C5—H5	127.9
Cl1—Cu1—Cl2	98.297 (16)	C4—C5—H5	127.9
C1—N1—N2	105.58 (12)	N4—C6—C5	107.30 (13)
C1—N1—Cu1	142.15 (11)	N4—C6—C7	121.67 (14)
N2—N1—Cu1	112.27 (8)	C5—C6—C7	131.03 (14)
C3—N2—N1	111.92 (13)	C6—C7—H7A	109.5
C3—N2—C4	132.08 (13)	C6—C7—H7B	109.5
N1—N2—C4	116.00 (10)	H7A—C7—H7B	109.5
C4—N3—N4	105.72 (11)	C6—C7—H7C	109.5
C4—N3—Cu1	119.46 (10)	H7A—C7—H7C	109.5
N4—N3—Cu1	134.50 (9)	H7B—C7—H7C	109.5
C6—N4—N3	111.41 (12)	C3—C8—H8A	109.5
C6—N4—H4	124.3	C3—C8—H8B	109.5
N3—N4—H4	124.3	H8A—C8—H8B	109.5
N1—C1—C2	109.39 (15)	C3—C8—H8C	109.5
N1—C1—C9	122.85 (15)	H8A—C8—H8C	109.5
C2—C1—C9	127.70 (14)	H8B—C8—H8C	109.5
C3—C2—C1	107.25 (13)	C1—C9—H9A	109.5
C3—C2—H2	126.4	C1—C9—H9B	109.5
C1—C2—H2	126.4	H9A—C9—H9B	109.5
N2—C3—C2	105.86 (14)	C1—C9—H9C	109.5
N2—C3—C8	123.69 (16)	H9A—C9—H9C	109.5
C2—C3—C8	130.41 (15)	H9B—C9—H9C	109.5

### Hydrogen-bond geometry (Å, °)

**Table:**

*D*—H···*A*	*D*—H	H···*A*	*D*···*A*	*D*—H···*A*
N4—H4···Cl1^i^	0.86	2.38	3.1587 (12)	150
C7—H7B···N1^ii^	0.96	2.61	3.483 (2)	151
C9—H9B···Cl1^iii^	0.96	2.79	3.5377 (19)	135

Symmetry codes: (i) −*x*, −*y*+1, −*z*; (ii) −*x*, −*y*+1, −*z*+1; (iii) −*x*+1, −*y*, −*z*.
